# Chimera states and collective chaos in pulse-coupled neural networks

**DOI:** 10.1186/1471-2202-12-S1-P336

**Published:** 2011-07-18

**Authors:** Simona Olmi, Antonio Politi, Alessandro Torcini

**Affiliations:** 1 CNR- Consiglio Nazionale delle Ricerche, Istituto dei Sistemi Complessi, Sesto Fiorentino, 50019, Italy; 2 INFN Sezione Firenze, via Sansone 1, Sesto Fiorentino, 50019, Italy; 3 Centro Interdipartimentale per lo Studio delle Dinamiche Complesse, via Sansone 1, Sesto Fiorentino, 50019, Italy

## 

Understanding the collective motion of networks of oscillators is crucial in many contexts, starting from neuronal circuits [1]. So far, most of the efforts have been devoted to the characterization of strong forms of synchronization. However, more subtle phenomena, like the onset of coherent oscillations in an ensemble of neurons can also play a relevant role for information coding. A peculiar coherent state, termed *Chimera*, appears in two symmetrically coupled populations of oscillators, where a population fully synchronizes while the other exhibit an asynchronous dynamics [2]. This can represents an idealized mathematical representation of the so-called unihemispheric sleep. Many creatures sleep with only half their brain at a time [3]. Such phenomenon was first reported in dolphins and other sea mammals and, recently, in birds; when brain waves are recorded, the awake side of the brain shows desynchronized electrical activity, corresponding to millions of neurons oscillating out of phase, whereas the sleeping side is highly synchronized.

In our study we have investigated the dynamics of two symmetrically coupled populations of identical leaky integrate-and-fire neurons characterized by excitatory coupling [4]. Upon varying the coupling strengths between and within the two populations, we found symmetry-breaking transitions that lead to the onset of various chimera states. To be more specific, in the observed chimera states one population is fully synchronized, while the other one is in the so-called Partially Synchronous (PS) regime. This regime is characterized by a coherent periodic activity at a collective level (somehow corresponding to to the Local Field Potential), while the single neurons behaves quasi-periodically [5]. To our knowledge this represents the first evidence of chimera states in pulse-coupled neural networks.

Furthermore a new regime, where the two populations are both PS but with a different degree of synchronization have been also observed. Even more interesting is the identification of a regions of parameters where the coherent activity of the network, characterized by collective variables, becomes chaotic. Collective chaos, meant as irregular dynamics of coarse-grained observables, has been found in ensembles of fully coupled one-dimensional maps as well as in two-dimensional continuous-time oscillators. In both classes of models, the single dynamical unit can behave chaotically under the action of a periodic forcing. Only a few examples of low-dimensional chaotic collective motion have been found in ensembles of phase oscillators because in this setup there is little space for a high-dimensional dynamics [6]. Our results reveal for the first time chaos at a macroscopic level in a neural network.

The computation of the the finite-amplitude Lyapunov exponent allows us to firmly establish the chaoticity of the (collective) dynamics in a finite region of the phase plane. The further numerical study of the standard Lyapunov spectrum reveals the presence of several positive exponents, indicating that the microscopic dynamics is high-dimensional.
